# Bidirectional Evolution of SARS‐CoV‐2 Virus in Immunocompromised Hosts

**DOI:** 10.1111/irv.13266

**Published:** 2024-03-10

**Authors:** Charmaine W. Y. Chew, Barnaby E. Young, Paul A. Tambyah, Shawn Vasoo, Conrad E. Z. Chan

**Affiliations:** ^1^ National Centre for Infectious Diseases Singapore Singapore; ^2^ Tan Tock Seng Hospital Singapore Singapore; ^3^ Lee Kong Chian School of Medicine Nanyang Technological University Singapore Singapore; ^4^ Division of Infectious Diseases, Department of Medicine National University Hospital Singapore Singapore; ^5^ Infectious Diseases Translational Research Program National University of Singapore Singapore Singapore; ^6^ Defence Medical and Environmental Research Institute DSO National Laboratories Singapore Singapore

To the editor:

SARS‐CoV‐2 has accumulated significant mutations to evade both vaccine‐ and infection‐induced immunity. Some of the currently dominant Omicron subvariants are thought to have initially evolved in immunocompromised patients despite the lack of selection pressure from the host immune response or poor vaccine response [1]. Interestingly, some of these mutations such as K417N and F486S have been shown to reduce spike affinity for the cellular ACE2 receptor and lead to reduced fitness in vitro [2, 3]. Consistent with these findings, we have observed the loss of the mutation F486S in 0.29% of XBB sequences from Singapore submitted to GISAID between October and December 2022. This reversion of acquired mutations has not been previously reported to our knowledge. Clinical and laboratory data were available for two immunocompromised patients (a renal transplant recipient and another with relapsed lymphoma) with such reversions.

Receptor binding region sequences were obtained by Sanger sequencing from nasopharyngeal swabs using previously published primers [4], and viral load was determined by RT‐PCR using the Roche COBAS SARS CoV‐2 test kit and anti‐spike antibody serology using the Elecsys Anti‐SARS‐CoV‐2 S test kit. The dates of sampling, viral load, and duration of treatment are shown in Figure 1 and patient details in Table 1.

**FIGURE 1:**
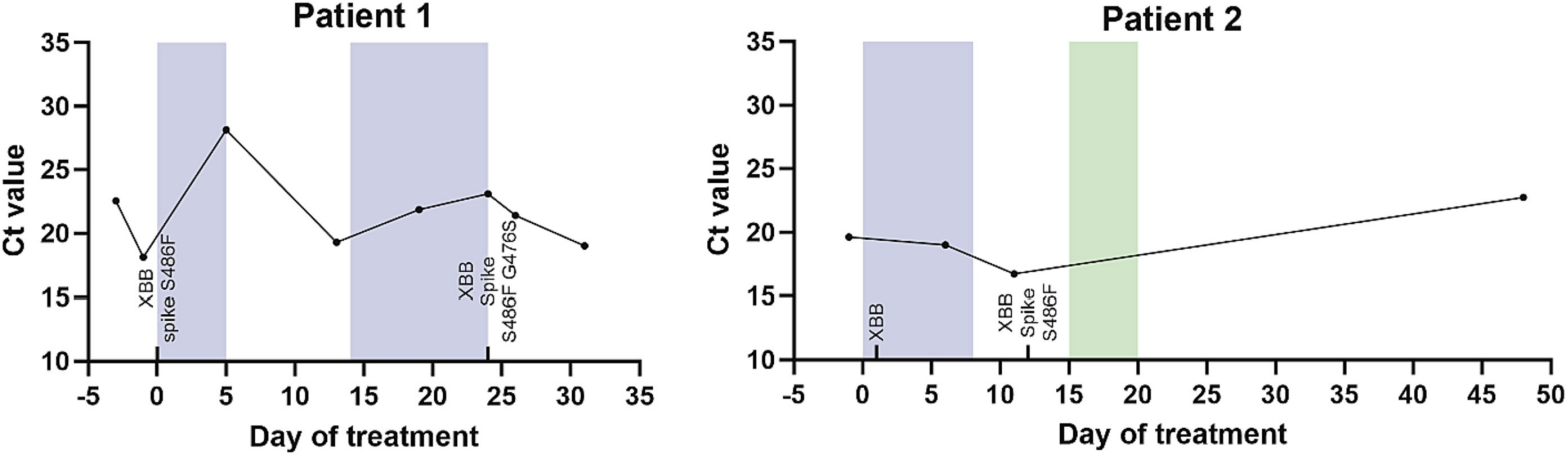
RBD mutations (indicated by PANGO variant and additional mutations, reversion is indicated as S486F), viral load (Ct values), and treatment received (blue: remdesivir, green: nirmatrelvir/ritonavir) for two immunocompromised patients. The timepoint of the sequence/viral load is indicated by day of treatment.

**TABLE 1:** Patient clinical details.

	Patient 1	Patient 2
Age	76	54
Date of admission (month/year)	November 2022	December 2022
Medical history	Kidney transplant recipient (2014)	Relapsed DLBCL, stage IV with extensive mediastinal involvement
Total anti‐spike (U/mL) at admission	477	88.7
COVID vaccination	Fully vaccinated (×3 Pfizer)	Fully vaccinated (×3 Pfizer)
Disease severity (on O_2_/admitted ICU)	Not on O_2_, not admitted to ICU	On O_2_ Days 2–3 of treatment, not admitted to ICU

Both patients were identified to have the XBB subvariant, the dominant circulating strain in Singapore at that time. At initial sampling, the first patient's sample already exhibited reversion of F486S, which was also present at the second sampling 25 days later by which time a second spike mutation G476S had emerged. The second patient lacked the reversion on initial sampling but was found to have the reversion at the second sampling 12 days later without further mutational changes. While we cannot conclusively demonstrate that the first patient's reversion occurred within the host, the low frequency of this mutation in sequenced SARS‐CoV‐2 XBB genomes in Singapore collected between October and December 2022 [5] (five out of 1712, GISAID EPI_SET_231120fh) suggests that this might indeed have evolved in the immunocompromised host as was documented in the second patient.

Repeated infection with newer Omicron variants may result in an increase in variant‐specific but paradoxically a loss of cross‐variant immunity [6]. Our observations suggest that re‐emergence of variants resembling wild type could occur in hosts without pre‐Omicron immunity, which may lead to the higher case‐fatality rates associated with pre‐Omicron strains [7]. Our findings highlight the importance of continued genomic surveillance especially in immunocompromised hosts for variants with pre‐Omicron characteristics that may affect vaccination strategies for this population.

## Author Contributions

**Charmaine W.Y. Chew:** Formal analysis; investigation. **Barnaby E. Young:** Funding acquisition; resources; writing—original draft. **Paul A. Tambyah:** Resources; writing—original draft; writing—review and editing. **Shawn Vasoo:** Funding acquisition; supervision; writing—original draft; writing—review and editing. **Conrad E.Z. Chan:** Formal analysis; funding acquisition; investigation; supervision; writing—original draft; writing—review and editing.

## Ethics Statement

Ethics approval was obtained from the National Healthcare Group DSRB under the following study: 2012/00917—A Multi‐centered Prospective Study to Detect Novel Pathogens and Characterize Emerging Infections.

## Conflicts of Interest

A/Prof. Young reports personal fees from AstraZeneca, Gilead, Pfizer, Sanofi‐Pasteur, and Moderna outside the submitted work. Prof. Tambyah reports personal fees from Moderna and Sanofi‐Pasteur outside the submitted work. Other authors declare no conflicts of interest.

### Peer Review

The peer review history for this article is available at https://www.webofscience.com/api/gateway/wos/peer‐review/10.1111/irv.13266.

## Data Availability

The data that support the findings of this study are available from the corresponding author upon reasonable request.
